# Effects of Perioperative Inflammatory Response in Cervical Cancer: Laparoscopic versus Open Surgery

**DOI:** 10.3390/jcm10184198

**Published:** 2021-09-16

**Authors:** Ji-Hoon Sim, Ju-Seung Lee, Dong-Min Jang, Hwa Jung Kim, Shin-Wha Lee, Hyun-Seok Cho, Woo-Jong Choi

**Affiliations:** 1 Asan Medical Center, Department of Anesthesiology and Pain Medicine, University of Ulsan College of Medicine, Seoul 05505, Korea; atlassjh@hanmail.net (J.-H.S.); christa416@naver.com (J.-S.L.); dmjang@amc.seoul.kr (D.-M.J.); woojongchoi@amc.seoul.kr (W.-J.C.); 2 Asan Medical Center, Department of Clinical Epidemiology and Biostatistics, University of Ulsan College of Medicine, Seoul 05505, Korea; hello.hello.hj@gmail.com; 3 Asan Medical Center, Department of Obstetrics and Gynecology, University of Ulsan College of Medicine, Seoul 05505, Korea; swhlee@amc.seoul.kr

**Keywords:** cervical cancer, laparoscopic radical hysterectomy, neutrophil to lymphocyte ratio, survival

## Abstract

There are few studies between postoperative neutrophil to lymphocyte ratio (NLR) and survival in cervical cancer. We compared postoperative changes in NLR according to surgical methods and analyzed the effect of these changes on 5-year mortality of cervical cancer patients. A total of 929 patients were assigned to either the laparoscopic radical hysterectomy (LRH) (*n* = 721) or open radical hysterectomy (ORH) (*n* = 208) group. Propensity score matching analysis compared the postoperative NLR changes between the two groups, and multivariate logistic regression analysis evaluated the association between NLR changes and 5-year mortality. Surgical outcomes between the two groups were also compared. In the LRH group, NLR changes at postoperative day (POD) 0 and POD 1 were significantly lower than in the ORH group after matching (NLR change at POD 0, 10.4 vs. 14.3, *p* < 0.001; NLR change at POD 1, 3.5 vs. 5.4, *p* < 0.001). In multivariate logistic regression analysis, postoperative NLR change was not associated with 5-year mortality (2nd quartile: OR 1.55, 95% CI 0.56–4.29, *p* = 0.401; 3rd quartile: OR 0.90, 95% CI 0.29–2.82, *p* = 0.869; 4th quartile: OR 1.40, 95% CI 0.48–3.61, *p* = 0.598), whereas preoperative NLR was associated with 5-year mortality (OR 1.23, 95% CI 1.06–1.43, *p* = 0.005). After matching, there were no significant differences in surgical outcomes between the two groups. There were significantly fewer postoperative changes of NLR in the LRH group. However, the extent of these NLR changes was not associated with 5-year mortality. By contrast, preoperative NLR was associated with 5-year mortality.

## 1. Introduction

Cervical cancer, predominantly caused by the human papillomavirus (HPV), is one of the most common gynecological cancers [1]. Cervical cancer ranked as the fourth most common cancer among females worldwide in 2018, with an incidence rate of 13.1 per 100,000 and high mortality rates [2,3]. HPV vaccination, early screening, chemotherapy, and radiation therapy have improved outcomes for cervical cancer; however, the surgical method remains the main treatment for early cervical cancer patients [4,5]. However, cervical cancer has a high recurrence rate even after surgery. According to a related study, postoperative recurrence rates of stage I B–II A stage and II B–IV A stage were 10%–20% and 50%–70%, respectively [6].

Recently, there have been several reports stating that immune function and inflammatory response in cervical cancer patients is associated with recurrence and survival rate [7,8]. In addition, among many inflammatory biomarkers, neutrophil to lymphocyte ratio (NLR) and platelet to lymphocyte ratio (PLR) were reported as predictors of cancer staging, recurrence, and survival outcomes in cervical cancer [9,10,11]. Many studies reported that preoperative NLR as a marker for predicting tumor progression and prognosis in cervical cancer as well as other visceral cancers [12,13,14,15,16,17]. Postoperative NLR has also been reported to predict survival and complications in some cancers [18,19,20]. However, there are few studies between postoperative NLR changes by surgical methods (laparoscopic vs. open surgery) and survival in cervical cancer.

Therefore, we compared the postoperative NLR changes between cervical cancer patients who underwent laparoscopic radical hysterectomy (LRH) and open radical hysterectomy (ORH), and analyzed the association between changes of this ratio and the patients’ 5-year mortality.

## 2. Methods

### 2.1. Study Design and Patient Population

This study was approved by the institutional review board (IRB) of the Asan Medical Center (protocol number: 2020-1779), and the requirement for written informed consent was waived by the IRB. We retrospectively reviewed the data from patients who were diagnosed with cervical cancer based on the International Classification of Diseases, tenth revision (ICD-10), in our medical center. These patients underwent ORH or LRH between June 2006 and February 2015. Adult female patients aged ≥ 18 years were included in the study. The exclusion criteria were as follows: (1) patients aged < 18 or ≥ 80 years, (2) patients who had severe diseases such as cardiovascular disease, hematologic disease, inflammatory disease, and other malignancies, (3) patients who have received any cervical neoplasia treatment prior to the admission, (4) patients who converted from laparoscopic surgery to laparotomy, and (5) patients with incomplete data from medical records.

### 2.2. Clinical Data Collection and Outcome Assessments

All patient data were obtained from the electronic medical record system, including demographic data, intraoperative variables, and laboratory values at preoperative and postoperative days (POD) 0 and 1. The demographic data included age, weight, height, body mass index (BMI), the American Society of Anesthesiologists (ASA) physical status classification, comorbidities, lymph node metastasis, postoperative chemotherapy, and radiation therapy. The histopathological records of the patients were examined and classified into six categories (1 = squamous cell carcinoma, 2 = adenocarcinoma, 3 = adenosquamous cell carcinoma, 4 = small-cell carcinoma, 5 = neuroendocrine carcinoma, and 6 = not reported). The cancer staging had been based on the International Federation of Gynecology and Obstetrics (FIGO) stage classification (stage I, II, III, and IV). Laboratory values of preoperative, POD 0, and POD 1 included white blood cell, hemoglobin, platelet count, total neutrophil count, total lymphocyte count, and serum albumin. Patients’ full blood counts were determined preoperatively < 2 days after admission and prior to treatment, in the ward immediately after surgery, and routinely at POD 1 for all patients. NLR was defined as the ratio between absolute neutrophil count to absolute lymphocyte count, and PLR was determined as the ratio between absolute platelet count to absolute lymphocyte count. The NLR values at preoperative, POD 0, and POD 1 were recorded for all patients. Intraoperative variables included operation time, transfusion, total infused fluids, and colloid use. Postoperative hospital stays, intensive care unit (ICU) admission, and 5-year mortality rate were also recorded.

The primary outcome was the comparison of postoperative NLR changes between the two groups and an assessment of the association between 5-year mortality and NLR changes. The secondary outcome was the comparison of surgical outcomes such as ICU admission and hospital stay between the two groups.

### 2.3. Statistical Analysis

Data are expressed as the mean (standard deviation), or number (proportion), as appropriate. The data variables included in this study were compared between the ORH and LRH groups using the independent t test or Mann–Whitney U test for continuous variables, or the Chi squared or Fisher’s exact test for categorical variables.

We used logistic regression analysis to identify the risk factors associated with 5-year mortality. All variables with *p*-values < 0.1 in the univariate analysis were included in the multivariate analysis. We also performed multivariable logistic regression analysis to determine the propensity score using the following 17 variables: age, height, weight, BMI, surgeons, diabetes mellitus (DM), hypertension (HTN), ASA classification, FIGO stage, lymph node metastasis, and preoperative laboratory values (hemoglobin, platelet count, total neutrophil count, total lymphocyte count, NLR, PLR, and serum albumin). After 1:1 propensity score matching, the final analysis included 160 patients each in the ORH and LRH groups. In all statistical analyses, *p*-values < 0.05 were considered significant. All data were analyzed using R (version 3.1.2; R Foundation for Statistical Computing, Vienna, Austria) and IBM SPSS (version 22; IBM Corp., Armonk, NY, USA).

## 3. Results

**Figure 1:**
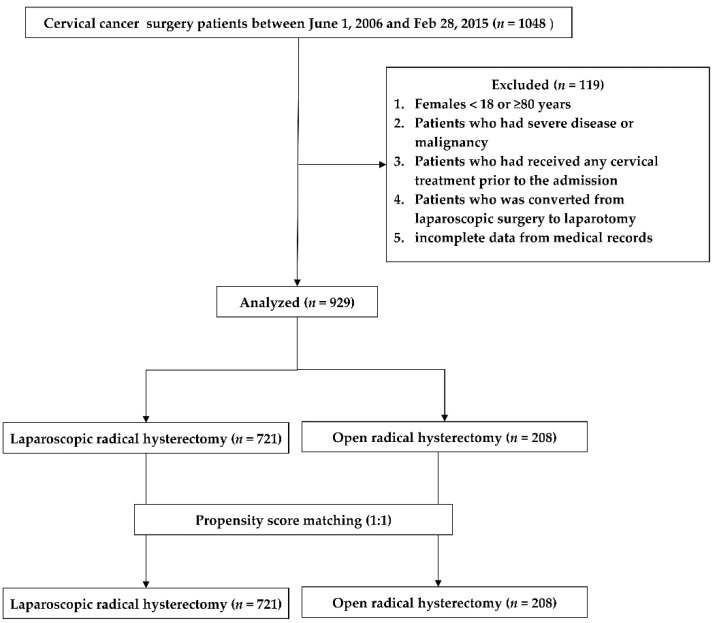
Study flow chart.

A total of 929 patients were enrolled in our study. Patients were divided into the LRH (*n* = 721) and ORH groups (*n* = 208), and their data were then analyzed after propensity score matching (Figure 1).

Table 1 shows the baseline characteristics and the perioperative variables of each group among unmatched and matched patients. Before the propensity score matching analysis, there were significant differences in age (*p* = 0.037), height (*p* = 0.034), surgeon (*p* < 0.001), FIGO stage (*p* < 0.001), lymph node metastasis (*p* < 0.001), preoperative white blood cell (*p* = 0.050), hemoglobin (*p* < 0.001), platelet count (*p* = 0.050), total neutrophil count (*p* < 0.001), total lymphocyte count (*p* = 0.002), NLR (*p* < 0.001), PLR (*p* = 0.001), and albumin (*p* < 0.001) between the two groups. By contrast, there were no significant differences in weight, BMI, DM, HTN, and ASA status. After the propensity score matching analysis, no significant differences in these variables were observed between the two groups. The intraoperative and postoperative variables of each group are also listed in Table 1. After matching, the LRH group received fewer transfusion (*p* < 0.001) and less postoperative radiation therapy (*p* = 0.001). Moreover, there were no significant differences in operation time, total infused fluid (mL/kg), colloid use, histology, and postoperative chemotherapy after matching between the two groups

### 3.1. Primary Outcomes

**Figure 2:**
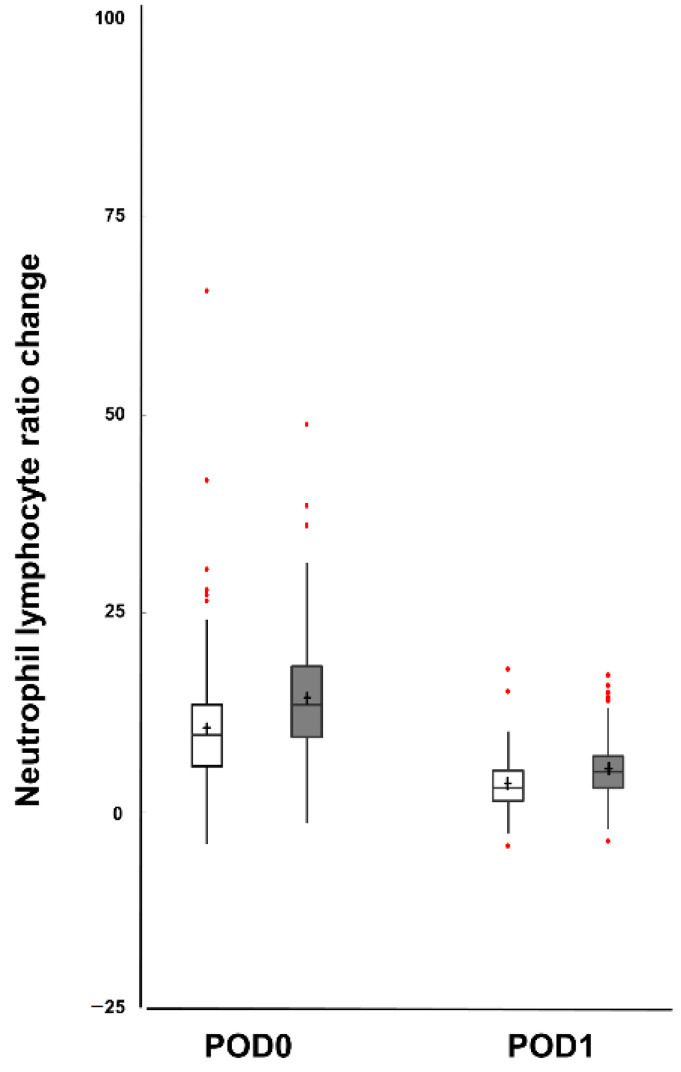
Comparison of the postoperative neutrophil to lymphocyte ratio (NLR) changes on postoperative days (POD) 0 and 1 between the laparoscopic radical hysterectomy group (LRH; white column) and open radical hysterectomy group (ORH; grey column). There were significant differences in the NLR changes between the two groups (*p* < 0.001 for all outcome variables). The central box represents the values from the lower to the upper quartile (25th to 75th percentile). The middle line represents the median. A line extends from the minimum to maximum value, excluding outliers. The cross sign and error bars of each group represent the means and standard deviations, respectively.

The postoperative NLR changes between the two groups after matching are shown in Figure 2. Significant differences were observed between the two groups (at POD 0 of NLR change, 10.4 vs. 14.3, *p* < 0.001; at POD 1 of NLR change, 3.5 vs. 5.4, *p* < 0.001) (Figure 2).

In the multivariate logistic regression analysis of risk factors for 5-year mortality, three significant factors were identified: small-cell carcinoma histology (OR 9.86, 95% CI 2.83–34.42, *p* < 0.001), postoperative chemotherapy (OR 15.21, 95% CI 2.95–78.48, *p* = 0.001), and preoperative NLR (OR 1.23, 95% CI 1.06–1.43, *p* = 0.005) (Table 2). By contrast, postoperative NLR changes at POD 0 were not associated with 5-year mortality (2nd quartile: OR 1.55, 95% CI 0.56–4.29, *p* = 0.401; 3rd quartile: OR 0.90, 95% CI 0.29–2.82, *p* = 0.869; 4th quartile: OR 1.40, 95% CI 0.48–3.61, *p* = 0.598) (Table 2).

### 3.2. Secondary Outcomes

Table 3 shows the comparison of the surgical outcomes between the LRH and ORH groups before and after matching. There were significant differences in hospital stay and overall mortality before matching (9.8 days vs. 12.4 days, *p* < 0.001, 3.9% vs. 8.2%, *p* = 0.011) (Table 3). However, after propensity scoring matching, no significant differences were observed in terms of surgical outcomes. The hospital stay was shorter in the LRH group than in the ORH group, but there was no statistical significance to this difference (10.6 days vs. 11.7 days, *p* = 0.080) (Table 3). Moreover, the 5-year mortality of the LRH group was not significantly different than that of the ORH group both before and after matching (3.2% vs. 5.8%, *p* = 0.085, 3.8% vs. 4.4%, *p* = 0.777) (Table 3). Similarly, the incidence of ICU admission was not significantly different between the LRH and ORH groups both before and after matching (1.2% vs. 1.5%, *p* = 0.696, and 0.7% vs. 1.3%, *p* = 0.562) (Table 3).

Laparoscopic surgery was not significantly associated with 5-year mortality even after adjusting for other potentially confounding variables, both before and after matching (OR 0.92, 95% CI 0.41–2.08, *p* = 0.848, and OR 1.02, 95% CI 0.44–2.38, *p* = 0.968, respectively) (Table 4).

## 4. Discussion

Our study demonstrated that the postoperative increase in the inflammatory biomarker NLR was lower in the LRH than in the ORH group. This suggests that laparoscopic surgery may be superior to open surgery in terms of the postoperative inflammatory response. However, there was no significant association between 5-year mortality and NLR changes. The risk factors associated with the 5-year mortality were preoperative NLR and histology. These results indirectly suggest that there is no significant relationship between postoperative inflammatory response and survival in cervical cancer patients.

Previous studies established that systemic inflammation, manifested as neutrophilia, thrombocytosis, and relative lymphocytopenia, is involved in cancer progression at different stages, such as initiation, promotion, invasion, and metastasis [21]. Neutrophils and platelets are reportedly involved in tumor progression by providing angiogenesis, epithelial and stromal growth factors, and matrix remodeling enzymes [22]. Recently, preoperative NLR and PLR have been investigated as independent prognostic factors that determine cancer progression and recurrence [23,24]. Additionally, postoperative NLR was reported as an independent risk factor of postoperative complications and survival in some cancers [18,19,20,25]. Surprisingly, few studies have reported an association between postoperative NLR changes and surgical outcomes for cervical cancer according to the surgical method. In one study of cervical cancer, laparoscopic surgery reportedly showed fewer cytokine changes within 5 days and, thus, lower surgical stress than laparotomy [26]. To our knowledge, this is the first study to evaluate the association between the postoperative NLR and survival in cervical cancer. Our current study may be clinically meaningful for cervical cancer patients because of our evaluation of postoperative changes in NLR according to two surgical methods, and our analysis of the association between 5-year mortality and the postoperative increase in NLR.

In this study, the small increase in NLR in laparoscopic surgery compared to open surgery is thought to be due to minimal incision and surgical manipulation, and this is associated with rapid recovery, low incidence of surgical site infections, and short hospital stay [27,28,29]. These results are consistent with a study that reported that laparoscopic surgery had less postoperative inflammatory responses than open surgery [30]. Although statistical significance was slightly insufficient in our study after matching, the results before and after matching demonstrated a shorter hospital stay in the LRH group than the ORH group, which is considered to be clinically meaningful. However, in the multivariate analysis, postoperative NLR changes did not affect the 5-year mortality. These results suggest two important findings: First, transient postoperative inflammatory response may be less associated with long-term mortality. Second, laparoscopic surgery has temporary advantages in postoperative inflammatory response, but does not seem to have a significant effect on long-term mortality in cervical cancer patients. Previous studies have shown that in early-stage cervical cancer, laparoscopic surgery has better surgical outcomes compared to open surgery [4,5]. However, a meta-analysis showed that there was no significant difference in 5-year mortality [31], and since a recent prospective randomized clinical trial showing that laparoscopic surgery had lower disease-free survival and overall survival rates than open surgery [32], most of the world’s guidelines have accepted that survival after laparoscopic surgery is worse than open surgery. The results of our study were consistent with the meta-analysis reporting that laparoscopic surgery did not show a significant difference in 5-year mortality compared to open surgery [31].

We determined that the factors associated with the 5-year mortality were not postoperative NLR changes but preoperative NLR in multivariate analysis. These results are consistent with many previous studies reporting that preoperative NLRs are associated with survival rates in cancer patients [9,10,11,13,14,16,17]. Another factor associated with the 5-year mortality is small-cell carcinoma histology, which reportedly has a very poor prognosis [33,34], and postoperative chemotherapy.

Nevertheless, this study has several limitations. First, the major limitations of this study are those inherent to a retrospective study. Thus, there is a possibility of potential biases associated with patient selection and recall. However, to make up for these shortcomings, we performed propensity score matching for 17 variables. Second, only the blood test values on POD 0 and POD 1 were included in this study. Therefore, a well-designed prospective study with laboratory tests performed over a long period of time is needed to determine how long surgical methods affect changes in NLR, and whether these affect long-term surgical outcomes. Third, our data are based on the information listed in medical records collected by a single medical center. Hence, there is a possibility of biased results due to similar or homogeneous groups.

## 5. Conclusions

In cervical cancer patients, LRH had less of an increase in postoperative NLR changes than ORH. However, these changes do not seem to have a significant effect on the 5-year mortality. The 5-year mortality might be associated with the preoperative NLR, histology, and postoperative chemotherapy, not the change and extent of the postoperative NLR.

## Figures and Tables

**Table 1:** Baseline characteristics of the study populations in the unmatched and matched samples.

	Unmatched Sample				Matched Sample			
	Unmatched Sample: Lapa (*n* = 721)	Unmatched Sample: Open (*n* = 208)	*p*	SMD	Matched Sample: Lapa (*n* = 160)	Matched Sample: Open (*n* = 160)	*p*	SMD
Preoperative data								
Age; year	47.20 ± 11.33	49.09 ± 11.89	0.037	0.162	48.14 ± 11.06	48.62 ± 11.91	0.708	0.042
Weight	57.80 ± 8.29	57.79 ± 8.75	0.982	−0.002	57.39 ± 7.89	57.88 ± 8.66	0.601	0.059
Height	157.36 ± 5.54	156.42 ± 5.84	0.034	−0.164	156.23 ± 5.61	156.31 ± 5.81	0.903	0.014
BMI; kg·m^−2^	23.36 ± 3.24	23.63 ± 3.48	0.300	0.080	23.52 ± 3.03	23.70 ± 3.42	0.614	0.056
DM	33 (4.6)	12 (5.8)	0.480	0.054	12 (7.5)	9 (5.6)	0.498	−0.076
HTN	89 (12.3)	33 (15.9)	0.185	0.101	26 (16.2)	24 (15.0)	0.758	−0.034
ASA status			0.363	0.101			0.900	0.052
ASA 1	182 (25.2)	50 (24.0)			42 (26.2)	43 (26.9)		
ASA 2	531 (73.6)	153 (73.6)			115 (71.9)	115 (71.9)		
ASA 3	8 (1.1)	70 (2.6)			3 (1.9)	2 (1.2)		
Surgeons			<0.001	0.814			0.969	0.083
Surgeon 1	247 (34.3)	14 (6.7)			15 (9.4)	13 (8.1)		
Surgeon 2	124 (17.2)	81 (38.9)			57 (35.6)	59 (36.9)		
Surgeon 3	114 (15.8)	32 (15.4)			22 (13.8)	24 (15.0)		
Surgeon 4	67 (9.3)	32 (15.4)			24 (15.0)	26 (16.2)		
Surgeon 5	169 (23.4)	49 (23.6)			42 (26.2)	38 (23.8)		
FIGO			<0.001				0.436	
Precancerous lesion	5 (0.7)	2 (1.0)			1 (0.6)	1 (0.6)		
Stage 1A1	48 (10.8)	8 (3.8)			8 (5.0)	6 (3.8)		
Stage 1A2	53 (7.4)	9 (4.3)			13 (8.1)	9 (5.6)		
Stage 1B1	418 (58.0)	79 (38.0)			69 (43.1)	72 (45.0)		
Stage 1B2	89 (12.3)	29 (13.9)			22 (13.8)	24 (15.0)		
Stage 2A	63 (8.7)	31 (14.9)			25 (15.6)	14 (8.8)		
Stage 2B	40 (5.5)	41 (19.7)			18 (11.3)	31 (19.4)		
Stage 3	3 (0.4)	2 (1.0)			2 (1.2)	1 (0.6)		
Stage 4	2 (0.3)	7 (3.4)			2 (1.2)	2 (1.2)		
Lymph node metastasis	144 (20.0)	58 (27.9)	0.015		33 (20.6)	39 (24.4)	0.423	
WBC, 10^3^/uL	6.35 ± 1.85	6.73 ± 2.69	0.050	0.142	6.33 ± 1.70	6.33 ± 1.94	0.993	0.013
Hemoglobin, g/dL	12.41 ± 1.32	11.93 ± 1.54	<0.001	−0.331	12.22 ± 1.31	12.12 ± 1.38	0.504	−0.075
Platelets, 10^9^/L	255.15 ± 61.87	265.10 ± 72.36	0.050	0.148	259.39 ± 61.65	260.04 ± 62.81	0.926	0.010
Neutrophil	2.21 ± 1.37	2.70 ± 2.40	<0.001	0.073	2.25 ± 1.48	2.29 ± 1.38	0.791	0.062
Lymphocyte	31.66 ± 9.58	29.24 ± 10.56	0.002	−0.240	31.25 ± 9.91	31.16 ± 9.93	0.939	−0.009
NLR	2.21 ± 1.37	2.70 ± 2.40	<0.001	0.249	2.25 ± 1.48	2.29 ± 1.38	0.791	0.030
PLR	146.86 ± 72.86	172.19 ± 105.10	0.001	0.304	136.75 ± 70.06	157.03 ± 78.13	0.580	0.023
Albumin; g·dL^−1^	4.02 ± 0.35	3.84 ± 0.40	<0.001	−0.469	3.91 ± 0.36	3.93 ± 0.34	0.645	0.052
Intraoperative data								
Transfusion	199 (27.6)	122 (58.7)	<0.001		47 (29.4)	85 (53.1)	<0.001	
RBC unit	0.60 ± 1.27	1.57 ± 1.96	<0.001		0.67 ± 1.42	1.35 ± 1.79	<0.001	
Operation time; min	284.76 ± 62.93	301.42 ± 61.16	0.001		302.32 ± 70.62	298.54 ± 61.05	0.609	
Total fluids; mL/kg	60.23 ± 23.77	68.20 ± 29.54	<0.001		65.41 ± 24.99	66.95 ± 27.99	0.605	
Colloid use	452 (62.7)	151 (72.6)	0.008		103 (64.4)	115 (71.9)	0.150	
Postoperative data								
NLR at POD 0	12.48 ± 6.76	17.06 ± 11.51	<0.001		12.70 ± 8.07	16.54 ± 7.43	<0.001	
NLR at POD 1	5.92 8.30	7.88 3.85	<0.001		5.84 6.42	7.93 5.61	<0.001	
Histology			0.009				<0.001	
Squamous	490 (68.0)	148 (71.2)			111 (69.4)	117 (73.1)		
Adeno	188 (26.1)	36 (17.3)			41 (25.6)	28 (17.5)		
Adenosquamous	27 (3.7)	17 (8.2)			6 (3.8)	11 (6.9)		
Small cell	9 (1.2)	6 (2.9)			1 (0.6)	4 (2.5)		
Neuroendocrine	1 (0.1)	0 (0.0)			1 (0.6)	0 (0.0)		
Not reported	6 (0.8)	1 (0.5)			0 (0.0)	0 (0.0)		
Postoperative CTx	267 (37.0)	124 (59.6)	<0.001		73 (45.6)	88 (55.0)	0.094	
Postoperative RTx	273 (37.9)	135 (64.9)	<0.001		74 (46.2)	103 (64.4)	0.001	

SMD, standardized mean difference; BMI, body mass index; DM, diabetes mellitus; HTN, hypertension; ASA, American Society of Anesthesiologists classification; FIGO, International Federation of Gynecology and Obstetrics; WBC, white blood cell; NLR, neutrophil to lymphocyte ratio; PLR, platelet to lymphocyte ratio; RBC, red blood cells; CTx, chemotherapy; RTx, radiation therapy. Values are expressed as the mean ± standard deviation, median (interquartile range), or *n* (proportion).

**Table 2:** Univariate and multivariate logistic regression analysis of 5-year mortality.

	Univariate			Multivariate		
	OR	95% CI	*p*-Value	OR	95% CI	*p*-Value
Age; year	0.98	0.95–1.01	0.156			
Weight	0.98	0.93–1.02	0.269			
Height	1.04	0.98–1.10	0.234			
BMI; kg·m^−2^	0.90	0.80–1.01	0.066			
DM	0.57	0.08–4.25	0.582			
HTN	1.11	0.42–2.91	0.837			
ASA			0.646			
ASA 1	1.00					
ASA 2	1.32	0.57–3.08	0.519			
ASA 3	2.68	0.30–23.56	0.374			
Surgeons			0.825			
Surgeon 1	1.00					
Surgeon 2	0.95	0.39–2.31	0.915			
Surgeon 3	0.74	0.25–2.13	0.572			
Surgeon 4	0.65	0.18–2.35	0.509			
Surgeon 5	0.59	0.22–1.59	0.295			
FIGO						
Precancerous lesion and stage 1	1.00			1.00		
Stage 2, 3, and 4	2.11	1.03–4.33	0.041	1.16	0.52–2.60	0.709
Lymph node metastasis	2.84	1.42–5.65	0.003	1.30	0.60–2.81	0.502
Transfusion	1.44	0.73–2.86	0.294			
Operation time; min	1.00	1.00–1.01	0.135			
Total fluids; mL/kg	1.01	1.00–1.02	0.115			
Colloids use	1.19	0.57–2.45	0.644			
Histology			<0.001			<0.001
Squamous	1.00			1.00		
Adeno	1.27	0.61–2.62	0.524	1.65	0.68–4.01	0.266
Adenosquamous	2.51	0.83–7.59	0.102	1.79	0.47–6.85	0.396
Small cell	11.15	3.60–34.51	<0.001	9.86	2.83–34.42	<0.001
Neuroendocrine and not reported	3.50	0.41–29.57	0.249	Infinite		0.998
NLR change at POD 0			0.741			0.868
1st quartile	1.00			1.00		
2nd quartile	1.06	0.42–2.66	0.898	1.55	0.56–4.29	0.401
3rd quartile	0.63	0.22–1.80	0.391	0.90	0.29–2.82	0.869
4th quartile	1.07	0.43–2.69	0.883	1.40	0.48–3.61	0.598
Albumin	0.36	0.16–0.81	0.014			
Preoperative NLR	1.28	1.14–1.45	<0.001	1.23	1.06–1.43	0.005
Preoperative PLR	1.02	1.01–1.03	<0.001			
Laparoscopic surgery	1.86	0.91–3.80	0.090	1.16	0.50–2.70	0.731
Postoperative CTx	12.49	4.89–31.91	<0.001	15.21	2.95–78.48	0.001
Postoperative RTx	4.37	2.19–8.72	<0.001	1.17	0.41–3.33	0.767

OR, odds ratio; CI, confidence interval; BMI, body mass index; DM, diabetes mellitus; HTN, hypertension; ASA, American Society of Anesthesiologists classification; FIGO, International Federation of Gynecology and Obstetrics; NLR, neutrophil to lymphocyte ratio; POD, postoperative day; CTx, chemotherapy; RTx, radiation therapy. Values are expressed as the mean ± standard deviation, median (interquartile range), or *n* (proportion).

**Table 3:** Surgical outcomes in the unmatched and matched samples.

	Unmatched Sample			Matched Sample		
	Unmatched Sample: Lapa (*n* = 721)	Unmatched Sample: Open (*n* = 208)	*p*	Matched Sample: Lapa (*n* = 160)	Matched Sample: Open (*n* = 160)	*p*
Hospital stay, day	9.8 ± 3.8	12.4 ± 7	<0.001	10.6 ± 4.8	11.7 ± 6.1	0.080
ICU admission	8 (1.2)	3 (1.5)	0.696	1 (0.7)	2 (1.3)	0.562
5-year mortality	23 (3.2)	12 (5.8)	0.085	6 (3.8)	7 (4.4)	0.777
Overall mortality	28 (3.9)	17 (8.2)	0.011	10 (6.3)	11 (6.9)	0.821

ICU, intensive care unit. Values are expressed as the mean ± standard deviation, median (interquartile range), or *n* (proportion).

**Table 4:** The 5-year mortality adjusted by laparoscopic surgery.

	Unmatched				Matched			
	Unadjusted OR (95% CI)	*p*	Adjusted OR * (95% CI)	*p*	Unadjusted OR (95% CI)	*p*	Adjusted OR ^†^ (95% CI)	*p*
5-year mortality	1.86 (0.91–3.80)	0.085	0.92 (0.41–2.08)	0.848	1.17 (0.39–3.57)	0.777	1.02 (0.44–2.38)	0.968

* Adjusted for age, BMI, operation time, histology, postoperative NLR change, albumin, postoperative chemotherapy, and radiation therapy. ^†^ Adjusted for FIGO stage, histology, postoperative NLR change, albumin, and preoperative NLR. OR, odds ratio; CI, confidence interval; BMI, body mass index; FIGO, International Federation of Gynecology and Obstetrics; NLR, neutrophil to lymphocyte ratio. Values are expressed as the mean ± standard deviation, median (interquartile range), or *n* (proportion).

## Data Availability

The dataset used and/or analyzed during the current study is available from the corresponding author upon reasonable request.
